# Noncontrast transcatheter aortic valve implantation technique with balloon-expandable prostheses

**DOI:** 10.1016/j.xjse.2024.100020

**Published:** 2024-08-25

**Authors:** Emre Polat, Anton Tomšič, Sina Stock, Evaldas Girdauskas, Tamer Owais

**Affiliations:** Department of Cardiothoracic Surgery, University Hospital Augsburg, Augsburg, Germany

**Figure:**
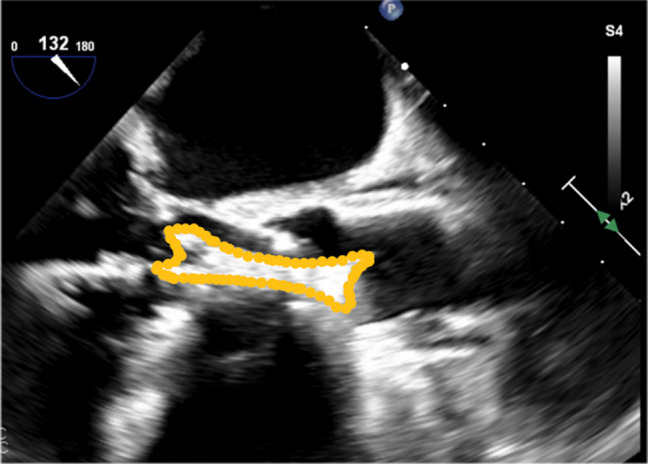
Dog bone configuration of a balloon-expandable valve prosthesis in fast-pacing phase.

**Central Message**

In this manuscript, we present a reproducible and feasible TAVI technique for patients with aortic valve stenosis who are unable to undergo contrast exposure. Our work underscores the critical need to continuously refine endovascular approaches for the aortic valve, showcasing alternative methods that hold promise for broader application.

The introduction of transcatheter aortic valve implantation (TAVI) signaled a paradigm shift for treating patients with severe aortic valve stenosis (AS). With growing expertise as well as continuous technical innovation, the procedure now presents a cornerstone in interventional treatment. Advanced fast-track pathways have further increased the safety of preprocedural, periprocedural, and postprocedural phases and reduced the median hospitalization time significantly.^1^ Recent studies have focused primarily on low and intermediate surgical risk patients with severe aortic valve stenosis.^2^ However, it is crucial to continue emphasizing complex and challenging TAVI procedures in high surgical risk patients through scientific inquiry, with the overarching goal of refining the procedure and enhancing outcomes for this patient cohort (Table E1).

Concerns persist regarding the administration of contrast agents in the elderly patients with underlying renal insufficiency, thyroid dysfunction, or a history of previous anaphylaxis to contrast solution exposure.^3^ Specifically, the occurrence of contrast-induced acute kidney injury (AKI) following computed tomography angiography for preinterventional planning and the TAVI procedure is a notable due to its association with increased morbidity and mortality.^4^ Ongoing endeavors are focused on devising efficient strategies to reduce the use of contrast in TAVI procedures through the application of diverse imaging modalities. Our aim here was to establish a safe interventional approach devoid of contrast agents, while ensuring favorable outcomes post-TAVI (Tables E2 and E3).

## Procedure

The diagnosis of AS is made by preoperative transthoracic echocardiography. Preoperative computed tomography scan of the aorta to iliofemoral arteries can be performed without angiography and can provide a gross impression of the arterial tree.

**Figure 1:**
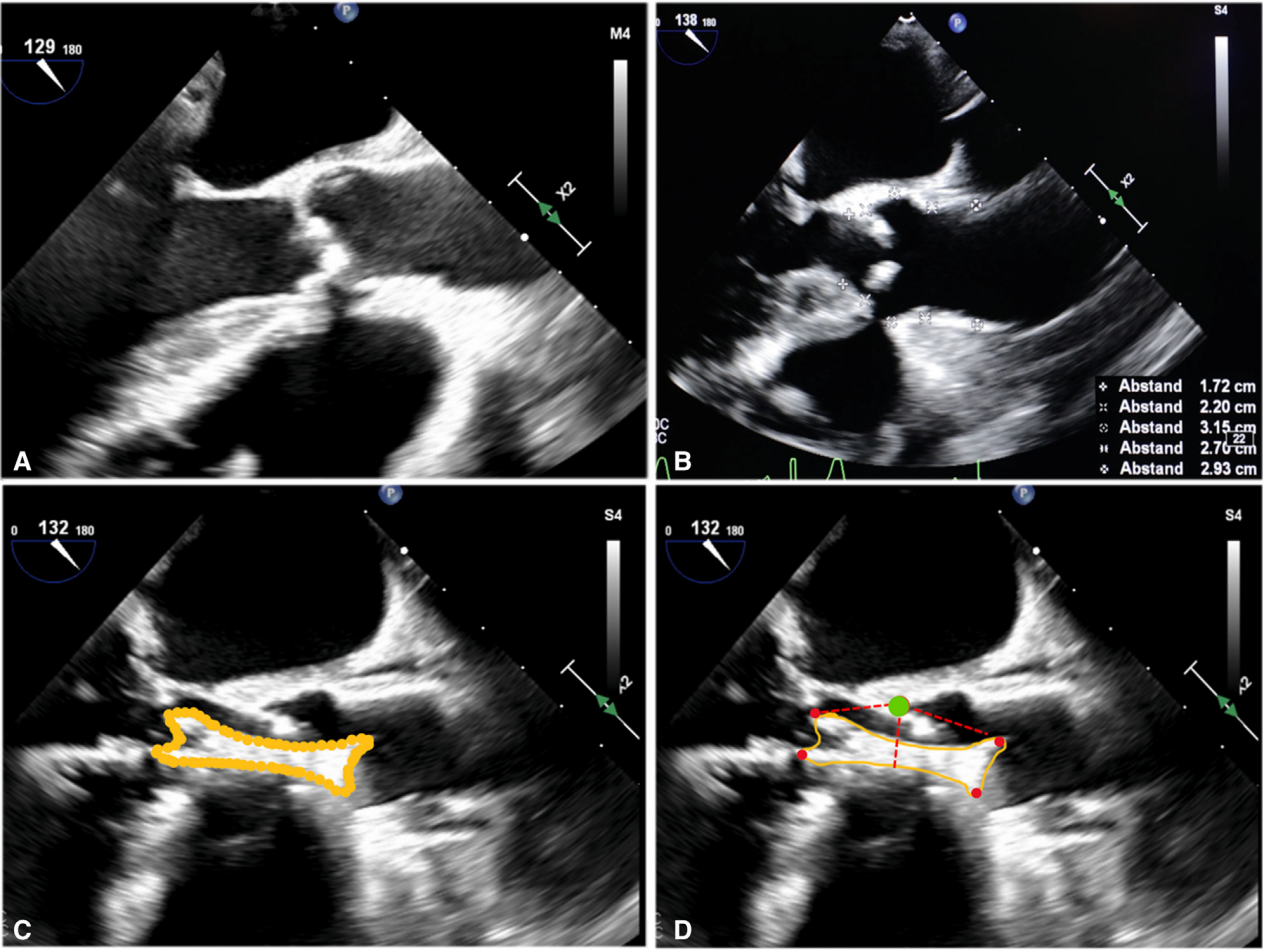
A, Midesophageal aortic valve, long-axis view with bulky calcification of the aortic cusps. B, Measured diameters of the left ventricular outflow tract, aortic annulus, sinus of Valsalva, sinotubular junction, and ascending aorta. C, Same view with a placed Safari guidewire in the left ventricle and a partially inflated (4 atm) ballon-expandable transcatheter valve prosthesis in a “dog bone” configuration serving as a reference for correct height positioning. D, Junction of the basal attachment of the aortic valvular leaflet (*green bullet*) as an anatomic landmark for optimal height positioning. For accurate deflation within the designated landing zone of the annulus, this critical anatomic landmark should be centered within the gradually inflated dog bone–shaped balloon-expandable prosthesis. The condyle-like configurations at both ends of the prosthesis (*red bullets*) serve as device-based landmarks that effectively illustrate the total frame height of the prosthesis.

The procedure is performed under general anesthesia and radiological and transesophageal guidance. Intraoperative duplex sonography is performed before each puncture to exclude plaques and transesophageal echocardiography (TEE) is performed for measuring annular dimensions and height positioning. Optimal alignment of the midesophageal aortic valve long-axis view in TEE is crucial for accurate sizing and height positioning, as it affords an exemplary perspective from the left ventricular outflow tract to the ascending aorta (Figure 1).

**Figure 2:**
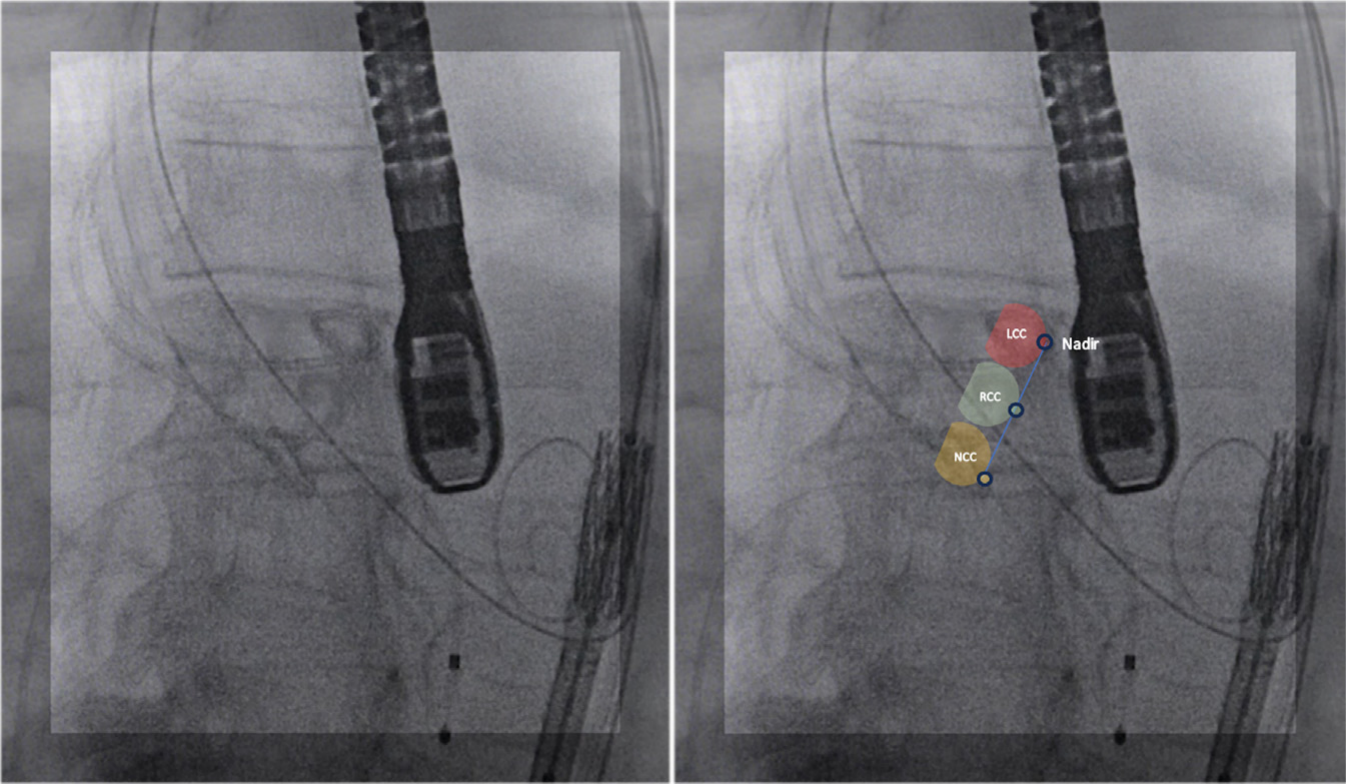
Positioning of 3 pigtails in the nadir of aortic valve cusps as a marker for annulus height.

First, the left femoral artery is punctured under sonographic guidance, and the first pigtail is positioned via a 5 Fr sheath in the noncoronary cusp. In cases where the fluorographic assessment of the annulus is limited owing to a lower calcific load of the native aortic valve, a second 5 Fr sheath is inserted through an additional ipsilateral puncture of the left femoral artery to place a second pigtail or guidewire in the right coronary cusp. To avoid parallax, an additional pigtail catheter is inserted through a third 5 Fr sheath via the right radial artery and positioned in the left coronary cusp (Figure 2).

Next, the procedure is continued in a standard fashion, placing a temporary pacemaker wire over the left femoral vein and introducing the TAVI delivery system via 14-French sheath in the right femoral artery. The implantation procedure is carried out in two distinct steps (Video 1). First, the balloon-expandable valve is positioned within the designated landing zone of the annulus. Subsequently, a “fast-pacing” phase is initiated at 140 bpm, with the balloon-expandable prosthesis gradually inflated to 4 atm. This step, while maintaining hemodynamic stability, leads to a “dog bone” configuration of the transcatheter valve prosthesis, as observed on intraoperative TEE, and allows for optimal height repositioning using the fine adjustment wheel if necessary (Figure 1).

Following the rapid pacing phase with 180 bpm in the second step, complete balloon inflation and deployment of the valve prosthesis in the predetermined position were carried out. In cases where significant paravalvular leakage was identified during intraoperative TEE, a postdilatation procedure could be performed safely. Coronary obstruction could be excluded by the absence of new regional wall motion abnormalities on TEE and the absence of new ischemic changes on electrocardiography. Assessment for femoral access site complications included a duplex ultrasound to rule out diminished blood flow.

## Discussion

Overall, our experience highlights the importance of tailored, safe alternative TAVI techniques for patients with distinct risk factors, such as underlying renal insufficiency. By refining the implantation steps and using meticulous imaging guidance, we avoid the use of contrast agents, with the primary aim of minimizing organ-specific stress, particularly on the kidneys.

Ferrari and colleagues^5^ reported on a case series of 30 patients who underwent the TAVI procedure via a transapical approach without the use of contrast media. In contrast, our technique focuses on the transfemoral approach, which is widely recognized as the primary approach and significantly less invasive.

Diaz and colleagues^6^ reported a single case of noncontrast TAVI procedure with a balloon-expandable prosthesis in a patient with the history of kidney transplant and a progressive decline in transplant function. The success of the case and in particular the technique were based solely on bulky calcifications of the aortic cusps as a reference on fluorography, and no additional safety techniques for correct height positioning were described. Busco and colleagues^7^ presented a successful case using a contrast-free TAVI technique involving the implantation of a self-expanding transcatheter valve prosthesis of the latest generation.

The literature contains very few descriptions of techniques including balloon-expandable valve prostheses. We believe that our technique is reproducible and successfully combines optimal height positioning of the balloon-expandable prosthesis with the known benefits of the transfemoral approach.

## Conclusions

Our proposed technique emphasizes the critical importance of further research and innovation in TAVI techniques to enhance patient outcomes and minimize complications, especially in challenging scenarios.

## Conflict of Interest Statement

The authors reported no conflicts of interest.

The *Journal* policy requires editors and reviewers to disclose conflicts of interest and to decline handling or reviewing manuscripts for which they may have a conflict of interest. The editors and reviewers of this article have no conflicts of interest.

## Figures and Tables

**Table E1:** Patient demographics

Characteristic	Patient												Median	SD
	1	2	3	4	5	6	7	8	9	10	11	12		
Age	81	83	77	77	78	84	61	79	75	80	80	79	79	±5.9
Sex	M	M	M	F	M	M	M	F	M	M	M	M		
Weight, kg	89	91	90	67	105	85	98	63	94	94	80	71	89.5	±12.9
Height, cm	181	178	172	164	183	184	180	163	181	186	179	183	180.5	±7.6
Body surface area, m^2^	2.1	2.1	2.0	1.7	2.3	2.1	2.2	1.7	2.2	2.2	2.0	1.9	2.1	±0.2
EuroScore II	6.4	4.1	5.2	6.2	6.2	7.3	13.1	4.5	4.1	5.2	4.7	6.1	5.7	±2.4
LVEF, %	40	43	39	40	35	45	25	45	40	39	40	38	40	±5.3
NYHA class	III	II	III	II	III	II	IV	II	II	III	II	III		
Hypertension	Y	Y	Y	N	Y	Y	Y	N	Y	N	N	Y	Y = 8	N = 4
Hyperlipidemia	Y	Y	N	Y	Y	N	Y	N	Y	Y	N	N	Y = 7	N = 5
COPD	Y	Y	Y	N	N	Y	Y	Y	Y	N	Y	Y	Y = 9	N = 3
Diabetes mellitus	Y	N	N	Y	Y	Y	Y	N	N	Y	N	Y	Y = 7	N = 5
Chronic kidney disease	Y	Y	Y	Y	Y	Y	Y	Y	Y	Y	Y	Y	Y = 12	N = 0
GFR, mL/min/1.73 m^2^	28	26	24	30	21	27	16	30	26	21	24	28	26.5	±4.3
Arrythmia	N	N	N	Y	Y	N	N	N	N	N	N	Y	Y = 3	N = 9
Tobacco use	Y	Y	Y	N	Y	Y	Y	Y	Y	Y	Y	Y	Y = 11	N = 1
Previous cardiac surgery	N	N	N	N	N	N	N	N	N	N	N	N	Y = 0	N = 12
Previous stroke	N	N	N	N	Y	N	Y	N	N	N	N	Y	Y = 3	N = 9
Coronary artery disease	N	N	N	N	N	N	N	N	N	N	N	N	Y = 0	N = 12
Myocardial infarction	N	N	N	N	N	N	N	N	N	N	N	N	Y = 0	N = 12

*LVEF*, Left ventricular ejection fraction; *NYHA*, New York Heart Association; *COPD*, chronic obstructive pulmonary disease; *GFR*, glomerular filtration rate.

**Table E2:** Procedural characteristics

Characteristic	Patient												Median	SD
	1	2	3	4	5	6	7	8	9	10	11	12		
Bicuspid valve morphology	N	N	N	N	N	N	N	N	N	N	N	N	79	±5.9
Symmetrical calcification	Y	Y	Y	Y	N	Y	Y	N	Y	Y	Y	N	Y = 9	N = 3
Prosthesis size, mm	26	26	23	23	26	29	26	23	29	29	26	23	26	±2.4
Horizontal aorta	N	N	N	N	N	N	N	N	N	N	N	N	Y = 0	N = 12
Predilatation	Y	Y	Y	Y	Y	Y	Y	Y	Y	Y	Y	Y	Y = 12	N = 0
Postdilatation	N	N	N	N	Y	N	Y	N	N	N	N	N	Y = 2	N = 10
Rapid pacing	Y	Y	Y	Y	Y	Y	Y	Y	Y	Y	Y	Y	Y = 12	N = 0
Procedure time, min:s	31:33	34:05	19:19	28:38	42:44	26:58	48:21	26:02	28:47	32:13	34:19	24:35	30:10	±05:49
Fluoro time, min:s	05:11	05:49	03:44	04:49	11:56	07:12	10:03	06:38	06:21	07:28	07:42	04:56	06:29	±01:59
On-table extubation	Y	Y	Y	Y	Y	Y	Y	Y	Y	Y	Y	Y	Y = 12	N = 0
FAST Track	Y	Y	Y	Y	Y	Y	Y	Y	Y	Y	Y	Y	Y = 12	N = 0

**Table E3:** Adverse events

Event	Patient												Total	
	1	2	3	4	5	6	7	8	9	10	11	12		
30-d mortality	N	N	N	N	N	N	N	N	N	N	N	N	Y = 0	N = 12
Valve embolization	N	N	N	N	N	N	N	N	N	N	N	N	Y = 0	N = 12
Conversion sternotomy	N	N	N	N	N	N	N	N	N	N	N	N	Y = 0	N = 12
Coronary obstruction	N	N	N	N	N	N	N	N	N	N	N	N	Y = 0	N = 12
Annular rupture	N	N	N	N	N	N	N	N	N	N	N	N	Y = 0	N = 12
Pericardial tamponade	N	N	N	N	N	N	N	N	N	N	N	N	Y = 0	N = 12
Paravalvular leakage	N	N	N	N	Y	N	N	N	N	N	N	N	Y = 1	N = 11
Worsened kidney injury	N	N	N	N	N	N	N	N	N	N	N	N	Y = 0	N = 12
Dialysis	N	N	N	N	N	N	N	N	N	N	N	N	Y = 0	N = 12
Major vascular complication	N	N	N	N	N	N	N	N	N	N	N	N	Y = 0	N = 12
Stroke	N	N	N	N	N	N	N	N	N	N	N	N	Y = 0	N = 12
Pacemaker implantation	N	N	N	Y	N	N	N	N	N	N	N	N	Y = 1	N = 11

## Supplementary Data

Video 1 Noncontrast transcatheter aortic valve implantation technique with a balloon-expandable prosthesis. https://www.jtcvs.org/article/S2950-6050(24)00020-2/fulltext.
