# Supplementary figures and images for: Noncontrast transcatheter aortic valve implantation technique with balloon-expandable prostheses
Source: JTCVS Struct Endovasc. 2024 Aug 25;3:100020. doi: 10.1016/j.xjse.2024.100020 (PMC13244745; doi:10.1016/j.xjse.2024.100020)

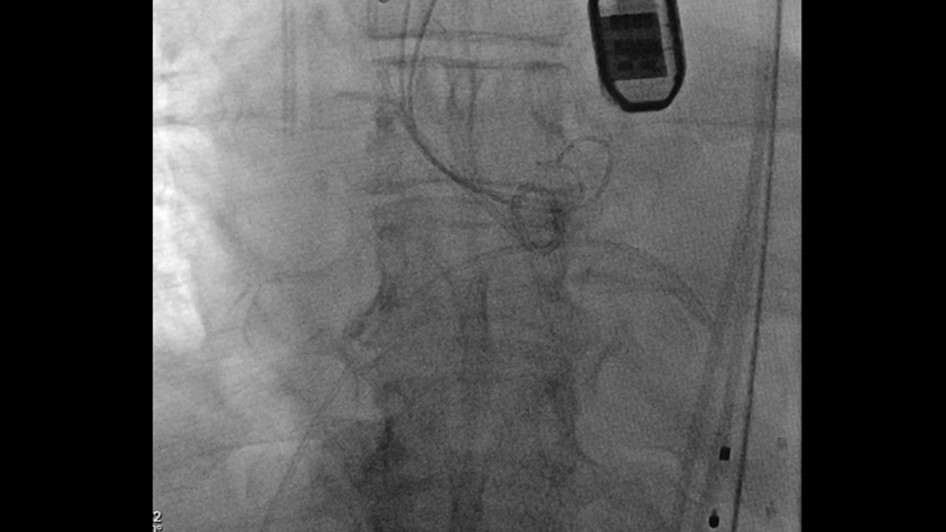

Supplement: Video 1 — Noncontrast transcatheter aortic valve implantation technique with a balloon-expandable prosthesis. https://www.jtcvs.org/article/S2950-6050(24)00020-2/fulltext. [file fx2.jpg]
